# Supplementary material for: Monitoring the Capacity of Microsporidia MB Transgenerational Spread in Anopheles arabiensis Populations
Source: Insects. 2025 Nov 27;16(12):1206. doi: 10.3390/insects16121206 (PMC12733993; doi:10.3390/insects16121206)
Supplement: Supplementary file 1 [file insects-16-01206-s001.zip › Additional File 1.pdf]

Table S1: The median absolute *Microsporidia MB* intensity across generations for replicate 1 and replicate 3.

Replicate	Generation	Median intensity	IQR
1	1	0.0715	8.3765
	2	0.1143	1.731
	3	0.228	4.8713
	4	1.3259	11.3174
	5	0.8947	16.8453
	6	0.0457	0.6344
3	1	0.0225	0.9542
	2	0.3382	3.3614
	3	0.0365	0.5307
	4	0.0048	0.2427
	5	0.0049	0.0074
	6	0.0037	0.0079

Table S2: Adjusted p-values for pairwise comparison of absolute *Microsporidia MB* intensity over generation in Replicate 1 and Replicate 3. Significant p-values are in bold.

Replicate	Generation	1	2	3	4	5
**1**	**2**	8.508				
	**3**	0.207	2.713			
	**4**	4.349	**0.001**	0.622		
	**5**	8.645	**0.003**	0.053	8.851	
	**6**	**0.000**	0.093	**0.002**	**0.000**	**0.000**
**3**	**2**	0.224				
	**3**	4.685	**0.006**			
	**4**	0.298	**0.004**	6.013		
	**5**	**0.001**	**0.000**	0.058	0.615	
	**6**	**0.003**	**0.000**	0.124	0.965	2.578

Table S3: The mean wing length size (S.D.) of female and male mosquitoes across generations in replicate 1.

Sex	Generation	Wing length	S.D.	n
**Females**	1	4.04	0.18	10
	2	3.68	0.18	9
	3	3.50	0.27	10
	4	3.59	0.26	10
	5	3.73	0.38	10
	6	3.59	0.13	10
**Males**	1	3.29	0.21	6
	2	3.16	0.21	8

	3	3.10	0.21	8
	4	2.95	0.23	10
	5	3.23	0.31	10
	6	3.24	0.18	10

Table S4: Adjusted p-values for pairwise comparison of winglength over generation in colony 1 for female and male mosquitoes. Significant p-values are in bold.

Sex	Generation	1	2	3	4	5
Female	2	0.050				
	3	**< 0.001**	1.000			
	4	**0.003**	1.000	1.000		
	5	0.122	1.000	0.684	1.000	
	6	**0.003**	1.000	1.000	1.000	1.000
Male	2	1.000				
	3	1.000	1.000			
	4	0.110	0.917	1.000		
	5	1.000	1.000	1.000	0.152	
	6	1.000	1.000	1.000	0.118	1.000
